# Construction and validation of a nomogram for predicting 3-month outcome in elderly patients with nonvalvular atrial fibrillation-induced acute ischemic stroke

**DOI:** 10.3389/fneur.2025.1561446

**Published:** 2025-05-09

**Authors:** Yang Yang, Xiaohong Zhang

**Affiliations:** Department of Cardiology, The First People’s Hospital of Hefei, The Third Affiliated Hospital of Anhui Medical University, Hefei, Anhui, China

**Keywords:** acute ischemic stroke, nonvalvular atrial fibrillation, prognosis, nomogram, clinical risk prediction model

## Abstract

**Aim:**

Cardiogenic embolism caused by atrial fibrillation (AF) leads to higher disability, mortality, and recurrence rates compared to stroke independent of AF, resulting in a poorer prognosis for patients. Effective risk assessment and timely clinical intervention are essential. This study aimed to develop and validate a personalized nomogram to predict the 3-month outcomes for elderly patients with nonvalvular atrial fibrillation (NVAF) induced acute ischemic stroke (AIS).

**Methods:**

A retrospective cohort study was implemented at Hefei First People’s Hospital. Participants were patients diagnosed with NVAF-induced acute ischemic stroke (NVAF-AIS) who fulfilled the study’s inclusion criteria. Data collection encompassed baseline demographic, clinical, and laboratory information. The primary endpoint was the 3-month outcome, evaluated using the modified Rankin Scale (mRS). To identify potential predictors, univariate logistic regression and the least absolute shrinkage and selection operator (LASSO) regression algorithm were employed. Subsequently, a binary regression model was established, and internal validation was conducted using bootstrap resampling with 1,000 iterations. The assessment tools included receiver operating characteristic (ROC) curves, calibration curves, and decision curve analysis (DCA). Ultimately, a nomogram was constructed to forecast the 3-month outcomes for this demographic.

**Results:**

A total of 178 patients were included, of whom 95 (53.3%) had a poor outcome (mRS > 2). Independent risk factors for poor outcomes in NVAF-AIS patients included stroke history (OR = 9.140; 95% CI: 3.481–26.923), NIHSS score (OR = 1.167; 95% CI: 1.071–1.284), glycated hemoglobin (HbA1c) (OR = 2.211; 95% CI:1.573–3.220), D-dimer (OR = 1.157; 95% CI: 1.022–1.361), neutrophil-to-lymphocyte ratio (NLR) (OR = 1.531; 95%CI:1.242–1.972), and left atrial diameter (LAD) (OR = 1.163; 95%CI: 1.072–1.280). A nomogram was created based on these factors. The area under the ROC curve (AUC) for the nomogram was 0.933 (95%CI:0.897–0.969) before and 0.933(95%CI:0.895–0.964) after internal validation, demonstrating good discriminative ability. The nomogram also showed excellent calibration and clinical applicability, as confirmed by calibration curve analysis and DCA.

**Conclusion:**

Stroke history, NIHSS score, HbA1c, D-dimer, NLR, and LAD are independent risk factors for poor outcomes in elderly patients with NVAF-AIS. The nomogram, integrating these factors, provides intuitive, individualized predictions for the risk of poor outcomes, aiding in the selection of treatment options for these patients.

## Introduction

Ischemic stroke (IS) represents the second most common cause of death and the third most common cause of disability globally, significantly impacting the economies of developing nations (1, 2). AF, a prevalent cardiac arrhythmia, is recognized as the primary contributor to increased cardiovascular mortality and a notable risk factor for stroke (3). In elderly patients with NVAF, the likelihood of stroke may increase fourfold to fivefold (4). AF-induced stroke typically presents with large, multiple, and bilateral cerebral infarcts. Furthermore, stroke associated with AF is often more severe than stroke independent of AF, leading to higher rates of disability, mortality, and recurrence (5, 6). This condition profoundly affects the quality of life of patients and imposes considerable economic and psychological strains on patients and their families alike. Several advancements, such as endovascular recanalization therapies including intravenous thrombolysis (IVT) and endovascular thrombectomy (EVT), have been adopted widely in clinical settings to enhance AIS patient outcomes (7, 8). However, many patients derive limited benefit from these therapies and experience poor outcomes. This may be due to the narrow therapeutic time window (4.5 h) after the onset of stroke and the low recanalization rate (approximately 20%) (9). Anticoagulation therapy also shows promise in reducing mortality in stroke patients with AF. Unfortunately, there remains a significant gap between guideline recommendations and clinical practice. Moreover, the effect of early oral anticoagulation on long-term functional outcomes in patients with varying stroke severity remains unclear. The risk factors for adverse outcomes associated with the early recovery phase following the onset of acute stroke in NVAF patients deserve early evaluation. Only when clinicians fully understand it will they be able to use early treatment protocols on such patients who are under their intensive medical management or monitoring, devise a strategy for the prevention and control of the disease, improve the prognosis, and reduce the mortality rate among the patients.

In recent years, various prognostic models for post-stroke outcome have also been presented, including a Totaled Health Risks in Vascular Events (THRIVE) score (10), the Acute Stroke Registry and Analysis of Lausanne (ASTRAL) score (11), and the mRS score (12), the latter being probably the most commonly used primary outcome measure applied in stroke clinical trials performed to date. It is simple and easy to interpret and, therefore, is very commonly used to assess functional outcome in AIS patients. However, these models cannot make individualized predictions for stroke associated with NVAF due to the complexity and specificity of the underlying mechanisms. Up to date, there is no effective prediction tool for outcomes in patients with NVAF-associated stroke.

A nomogram is a graphical tool that can precisely calculate the risk probability of a clinical event by combining significant risk factors (13). It has been widely used to predict outcomes in various diseases, including IS. In this study, a cohort study was conducted with NVAF-AIS patients as participants. Clinical, laboratory, imaging, and 3-month follow-up data were collected, and a nomogram for predicting outcomes was developed. This study may provide valuable evidence for future clinical decision-making and treatment, ultimately aiming to improve patient outcomes.

## Methods

### Study population

**Figure 1:**
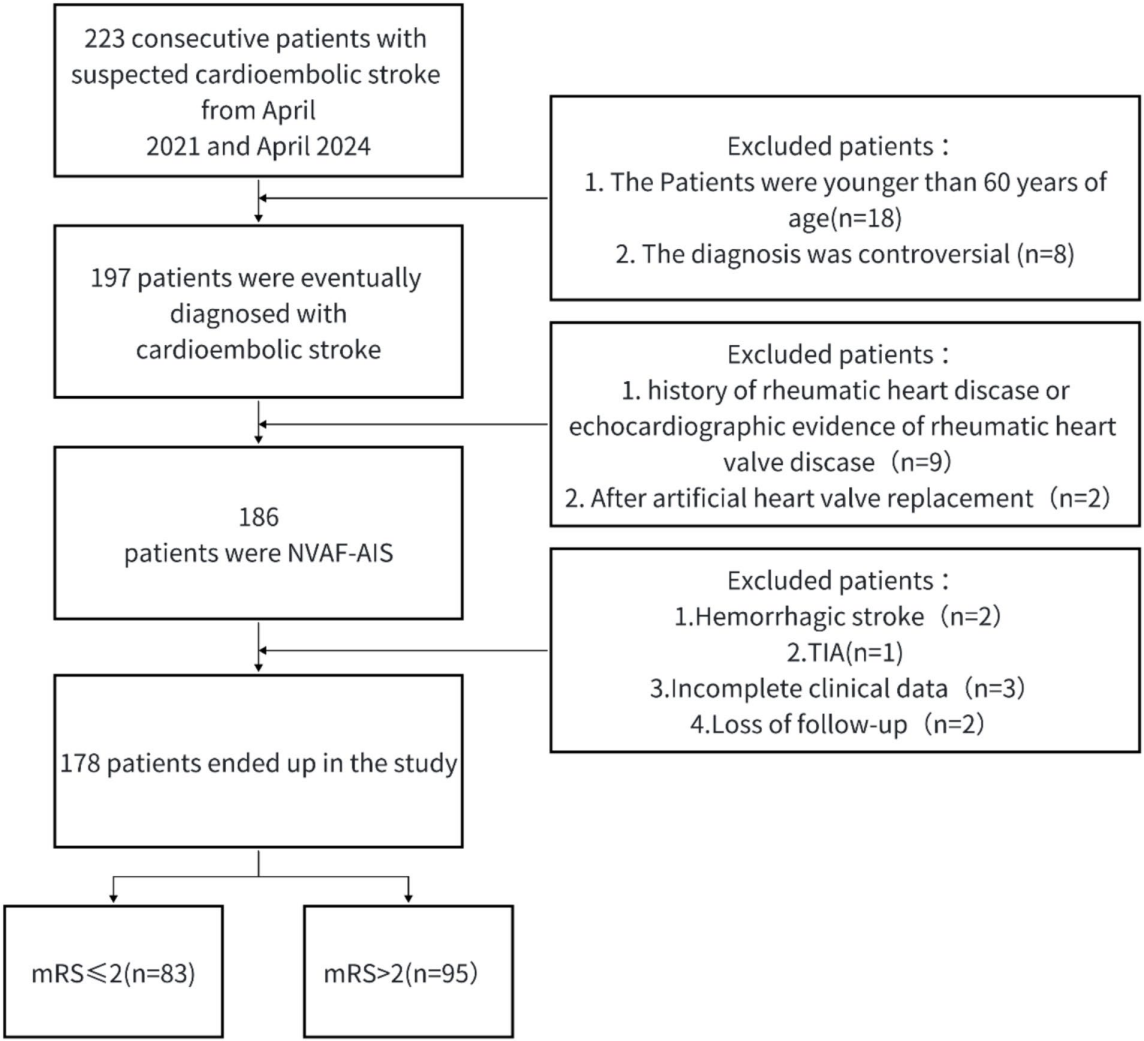
Flow chart of the study selection. NVAF-AIS, nonvalvular atrial fibrillation induced acute ischemic stroke; TIA, transient ischemic attack.

This single-center, retrospective, observational clinical study involved NVAF-AIS patients experiencing their first onset. From April 1, 2022, to April 1, 2024, 178 patients over 60 years old were recruited at Hefei First People’s Hospital. Baseline and follow-up data were collected and analyzed. Investigators were blinded to the study objectives and design during data collection. Inclusion criteria: (1) AF patients aged over 60 years; (2) AIS diagnosed by neurologists according to the Trial of Org 10,172 in Acute Stroke Treatment (TOAST) criteria (14). Exclusion criteria: (1) Hemorrhagic stroke; (2) Lacunar cerebral infarction or transient ischemic attack; (3) History of rheumatic heart disease or echocardiographic evidence of rheumatic valve disease; (4) History of prosthetic heart valve surgery; (5) history of malignancy; (6) incomplete clinical data; (7) loss to follow-up. The study received approval from the Ethics Committee of the hospital, and all participants gave informed consent, as depicted in the study flowchart (Figure 1).

### Baseline data

Baseline demographic and clinical data, including age, gender, smoking history, alcohol consumption, complications, AF type, and medication history, were collected. Additional data included CHA2DS2-VASc and HAS-BLED scores. Admission blood pressure and fasting laboratory markers were collected. Inflammatory indicators were also obtained. Echocardiographic parameters included LAD, left ventricular end-systolic diameter (LVESD), and left ventricular ejection fraction (LVEF).

AF patients in this study were those diagnosed with AF before the onset of stroke, according to the 2024 Chinese Guidelines on Diagnosis and Management of AF. Patients diagnosed with AF during the stroke (first onset) were excluded, as their stroke was believed to be caused by other factors, which later led to AF due to physiological stress. All patients with cardioembolic stroke were assessed by cardiology specialists. Based on the source of the cardioembolic thrombus, the patients were categorized into the following types: (a) structural heart diseases associated with arrhythmias, such as hypertensive heart disease with ventricular hypertrophy and rheumatic mitral valve disease; (b) structural heart disease with persistent sinus rhythm, such as systolic left ventricular dysfunction due to ischemic or non-ischemic causes; (c) isolated atrial arrhythmias, such as atrial fibrillation and atrial flutter (15). The final cohort of this study included patients with cardioembolic stroke caused by non-valvular structural heart disease associated with atrial fibrillation.

### Outcome indicator

Patients were classified into two groups based on their 3-month mRS scores: those with a good outcome (mRS ≤ 2, *n* = 83) and those with a poor outcome (mRS > 2, *n* = 95). Patients in this study were previously diagnosed with AF, according to the 2024 Chinese Guidelines on Diagnosis and Management of AF, prior to the onset of stroke. Factors associated with the outcome were analyzed. Investigators were blinded during data collection to ensure unbiased results.

### Statistical analysis

All statistical analyses were conducted using R 4.4.1. Continuous variables adhering to a normal distribution were presented as means ± standard deviations (SD) and analyzed using the independent samples *t*-test. Those not following a normal distribution were expressed as medians (P25, P75) and analyzed with the rank sum test. Categorical data were presented as frequencies (%) and analyzed using the Chi-square test or Fisher’s exact test as appropriate. Missing data were addressed through multiple imputation using the R package “missRanger.”

Univariate logistic regression was utilized to explore potential predictors of patient outcomes (*p* < 0.1). This was followed by LASSO regression to refine the selection of significant predictive factors, utilizing nonzero regression coefficients. The optimal model parameters, including the penalty parameter lambda, were defined through 10-fold cross-validation based on the minimum criteria. Subsequent analysis involved stepwise multiple logistic regression, leading to the construction of a nomogram for variables significant at *p* < 0.05. Internal model validation was performed using bootstrap resampling with 1,000 iterations. Model calibration was examined via a calibration curve, and the Hosmer-Lemeshow test assessed model fit. The discriminative ability of the model was evaluated by plotting a ROC curve and calculating the AUC, sensitivity, and specificity. Decision curve analysis (DCA) was applied to measure the clinical utility of the model across various decision thresholds.

## Results

### Baseline and laboratory data between two groups

Initially, 223 patients with cardiogenic stroke were recruited; of these, 178 were included in the final analysis as shown in Figure 1. Classification based on the 3-month mRS scores identified 95 patients (53.30%) in the poor outcome group and 83 (46.70%) in the good outcome group. Baseline characteristics of both groups are detailed in Table 1. Patients in the poor outcome group were generally older, had a history of stroke, more frequently used antiplatelet drugs, and exhibited higher CHA2DS2-VASc and NIHSS scores. Additionally, they had elevated levels of HbA1c, D-dimer, NC, LC, NLR, and LAD compared to those in the good outcome group (*p* < 0.05). Conversely, levels of HDL-C were significantly lower in the poor outcome group (*p* < 0.05). No significant differences in other variables were observed between the groups (*p* > 0.05).

**Table 1:** Comparison of baseline data between poor prognosis group and good prognosis group.

Variables	Poor prognosis group (*n* = 95)	Good prognosis group (*n* = 83)	*p*
Male [n(%)]	46 (48.4)	47 (56.6)	0.274
Age (years)	82.00 (73.00, 87.00)	78.00 (69.00, 82.50)	0.005
Smoking [n(%)]	30 (31.6)	26 (31.3)	0.971
Drinking [n(%)]	16 (16.8)	19 (22.9)	0.311
Hypertension [n (%)]	66 (69.5)	61 (73.5)	0.554
Coronary heart disease [n (%)]	20 (21.1)	17 (20.5)	0.925
Heart failure [n (%)]	36 (37.9)	21 (25.3)	0.072
Diabetes [n (%)]	31 (32.6)	19 (22.9)	0.149
History of stroke [n (%)]	61 (64.2)	19 (22.9)	< 0.001
Type of atrial fibrillation			0.784
Paroxysmal	37 (38.9)	34 (41.0)	
Persistent	58 (61.1)	49 (59.0)	
Systolic pressure (mmHg)	139.00 (126.00, 163.00)	134.00 (122.50, 150.00)	0.119
Diastolic pressure [n(%)]	81.00 (71.50, 91.00)	80.00 (72.50, 89.00)	0.727
Antiplatelet drug [n (%)]	28 (29.5)	14 (16.9)	0.048
Anticoagulant drugs [n (%)]	21 (22.1)	20 (24.1)	0.753
Antihypertensive drugs [n (%)]	57 (60.0)	49 (59.0)	0.896
CHA2DS2-VASc score	5.00 (5.00, 6.00)	5.00 (4.00, 6.00)	0.033
HAS-BLED score	3.00 (2.00, 3.00)	3.00 (2.00, 3.00)	0.935
NIHSS score	9.00 (6.00, 14.00)	4.00 (2.00, 8.50)	< 0.001
TG (mmol/L)	3.68 (3.16, 4.50)	3.93 (3.31, 4.86)	0.199
TC (mmol/L)	1.19 (0.88, 1.75)	1.15 (0.83, 1.61)	0.314
HDL-C (mmol/L)	1.16 (0.99, 1.41)	1.29 (1.02, 1.65)	0.032
LDL-C (mmol/L)	2.10 (1.59, 2.67)	1.96 (1.52, 2.49)	0.366
Lp(a) (mg/L)	215.59 (113.07, 372.06)	212.60 (123.13, 453.18)	0.670
HCY (μmol/L)	13.74 (11.59, 17.42)	14.25 (10.93, 19.18)	0.756
FBG (mmol/L)	5.66 (4.90, 6.79)	5.21 (4.54, 6.20)	0.117
UA (μmol/L)	321.11 ± 76.80	342.06 ± 94.62	0.110
SCR (μmol/L)	77.00 (68.50, 89.50)	80.00 (72.00, 95.50)	0.107
GFR (mL/min)	69.22 ± 15.57	68.86 ± 16.31	0.883
HbA1C (%)	7.80 (6.05, 8.75)	5.80 (5.44, 6.31)	< 0.001
D-dimer (mg/L)	2.08 (0.89, 6.38)	0.53 (0.26, 1.08)	< 0.001
NC (×10^9^/L)	4.72 (3.50, 6.54)	3.74 (3.17, 4.65)	< 0.001
LC (×10^9^/L)	1.15 (0.85, 1.58)	1.59 (1.28, 1.89)	< 0.001
PLT (×109/L)	164.00 (140.50, 198.50)	167.00 (146.00, 191.00)	0.839
HCT (%)	40.98 ± 4.29	40.95 ± 5.28	0.975
RDW (%)	13.50 (12.85, 14.10)	13.20 (12.75, 13.85)	0.132
NLR	4.07 (2.68, 6.76)	2.33 (1.71, 3.94)	< 0.001
PLR	132.99 (93.88, 187.14)	123.42 (88.55, 173.37)	0.230
LAD (mm)	45.00 (42.00, 48.00)	43.00 (39.00, 46.00)	0.005
LVEDD (mm)	49.00 (47.00, 51.00)	49.00 (47.00, 52.00)	0.376
LVEF (%)	63.00 (58.00, 66.50)	62.00 (57.50, 66.50)	0.914

NIHSS, the National Institute of Health Stroke Scale score; TG, triglyceride; TC, total cholesterol; LDL-C, low-density lipoprotein cholesterol; HDL-C, high-density lipoprotein cholesterol; Lp(a), lipoprotein(a); HCY, homocysteine; FBG, fasting blood glucose; SCr, serum creatinine; GFR, glomerular filtration rate; HbA1C, glycosylated hemoglobin, Type A1C; NC, neutrophil count; LC, lymphocyte count; PLT, platelet count; HCT, hematocrit; RDW, red cell distribution width; NLR, neutrophil-to-lymphocyte ratio; PLR, platelet to lymphocyte ratio; LAD, left atrial diameter; LVEDD, left ventricular end-diastolic diameter; LVEF, left ventricular ejection fraction.

### Univariate logistic regression analysis

Univariate logistic regression was utilized to pinpoint potential predictors of outcomes within this population (Table 2). Identified potential risk factors included age, heart failure history, previous strokes, systolic blood pressure, usage of antiplatelet drugs, CHA2DS2-VASc and NIHSS scores, HbA1c, D-dimer levels, NC, LC, NLR and LAD. Conversely, HDL-C was recognized as a potential protective factor.

**Table 2:** Univariate logistic regression analysis of factors affecting the prognosis of elderly NVAF-AIS.

Variable	β	SE	z	OR(95%CI)	*p*
Age (years)	0.042	0.016	2.573	1.043 (1.011, 1.078)	0.010
Heart failure [n (%)]	0.589	0.329	1.787	1.801 (0.951, 3.474)	0.074
History of stroke [n(%)]	1.799	0.338	5.327	6.043 (3.167, 11.948)	<0.001
Systolic pressure (mmHg)	0.011	0.006	1.717	1.011 (0.999, 1.024)	0.086
Antiplatelet drug [n (%)]	0.723	0.370	1.955	2.060 (1.012, 4.347)	0.051
CHA2DS2-VASc score	0.281	0.118	2.382	1.324 (1.056, 1.680)	0.017
NIHSS score	0.155	0.033	4.720	1.168 (1.098, 1.250)	<0.001
HDL-C (mmol/L)	−1.092	0.454	−2.402	0.336 (0.135, 0.807)	0.016
HbA1C (%)	0.792	0.142	5.588	2.207 (1.698, 2.967)	<0.001
D-dimer (mg/L)	0.268	0.071	3.791	1.308 (1.155, 1.528)	<0.001
NC (×109/L)	0.254	0.080	3.176	1.289 (1.113, 1.523)	0.001
LC (×109/L)	−1.056	0.281	−3.756	0.348 (0.195, 0.587)	<0.001
NLR	0.398	0.086	4.624	1.488 (1.271, 1.781)	<0.001
LAD (mm)	0.087	0.027	3.224	1.090 (1.037, 1.153)	0.001

*p < 0.1. NIHSS, the National Institute of Health Stroke Scale score; HDL-C, high-density lipoprotein cholesterol; HbA1C, glycosylated hemoglobin, Type A1C; NC, neutrophil count; LC, lymphocyte count; NLR, neutrophil-to-lymphocyte ratio; LAD, left atrial diameter.

### LASSO regression analysis

**Figure 2:**
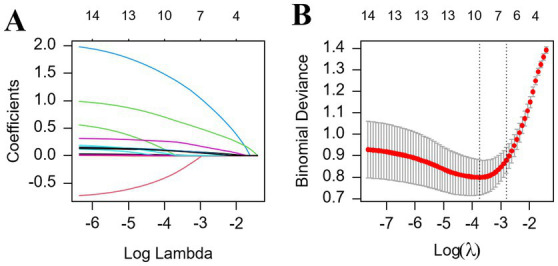
**(A,B)** Predictor plots screened by least absolute shrinkage and selection operator (LASSO) regression analysis. **(A)** Coefficient profiles were generated from the log(lambda) series. **(B)** In LASSO regression, the regularization parameter (lambda) for bias was selected using the minimum criterion (left dashed line) and the 1-SE criterion (right dashed line). In this study, predictors were chosen based on the 1-SE criterion (right dashed line). The optimal result is represented by the features with five non-zero coefficients. LASSO, least absolute shrinkage and selection operator; SE, standard error.

The 14 variables selected by univariate regression were subjected to LASSO regression analysis, which identified 10 significant variables with prognostic value. Age, heart failure history, previous strokes, NIHSS score, HDL-C, HbA1c, D-dimer, NC,NLR, and LAD were predictors of poor outcomes in elderly patients with NVAF-AIS (Figures 2A,B).

### Multivariate logistic regression analysis

The 10 significant variables were incorporated into a multivariate regression model. Previous strokes, NIHSS score, HbA1c, D-dimer, NLR, and LAD were independent predictors of poor outcomes in elderly patients with NVAF-AIS (*p* < 0.05, Table 3). All variables had a variance inflation factor (VIF) lower than 1.2 and a tolerance less than 0.7, indicating no collinearity between them.

**Table 3:** Multivariate logistic regression analysis of factors affecting the prognosis of elderly NVAF-AIS.

Variable	β	SE	OR(95%CI)	*p*
History of stroke [n(%)]	2.213	0.517	9.140 (3.481, 26.923)	<0.001
NIHSS score	0.155	0.046	1.167 (1.071, 1.284)	0.001
HbA1C (%)	0.793	0.181	2.211 (1.573, 3.220)	<0.001
D-dimer (mg/L)	0.146	0.072	1.157 (1.022, 1.361)	0.043
NLR	0.426	0.117	1.531 (1.242, 1.972)	<0.001
LAD (mm)	0.151	0.045	1.163 (1.072, 1.280)	0.001

*P < 0.05. NIHSS, the National Institute of Health Stroke Scale score; HbA1C, glycosylated hemoglobin, Type A1C; NLR, neutrophil-to-lymphocyte ratio; LAD, left atrial diameter.

### Nomogram construction and validation

**Figure 3:**
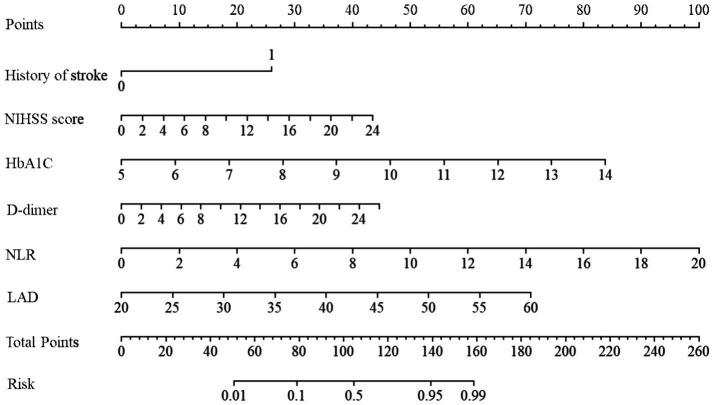
Nomogram for predicting the probability of 3-month unfavorable outcome in patients with Elderly NVAF - AIS. The nomogram incorporates variables such as history of stroke, NIHSS score, HbA1c, D-dimer, NLR, and LAD. The process for constructing the nomogram is as follows: First, for each variable in AIS patients, locate the corresponding score on the points line at the top. Next, sum all the scores and find the corresponding point on the total points line. Finally, identify the predicted probability corresponding to the patient on the predicted value line. NIHSS, the National Institute of Health Stroke Scale score; HbA1C, glycosylated hemoglobin, Type A1C; NLR, neutrophil-to-lymphocyte ratio; LAD, left atrial diameter.

The logistic regression analysis revealed six independent risk factors associated with poor outcomes in elderly patients with NVAF-AIS: history of stroke, NIHSS score, HbA1c, D-dimer, NLR, and LAD. These six variables were incorporated into a nomogram, with each assigned a specific score on the x-axis. An overall risk score was calculated by summing the individual scores of these variables. A higher overall score corresponded to a higher risk of poor outcome, while a lower score indicated a lower risk (Figure 3). In one NVAF-AIS patient with a history of stroke, the scores assigned to stroke history, NIHSS score (9), HbA1c (7.6), D-dimer (1.6), NLR (5.4), and LAD (38) were 27, 16, 24, 2.5, 25, and 32.5, respectively. The overall score was 127. According to the nomogram, the probability of poor outcome for this patient was approximately 85%, indicating a high risk of poor outcome (Figure 3).

**Figure 4:**
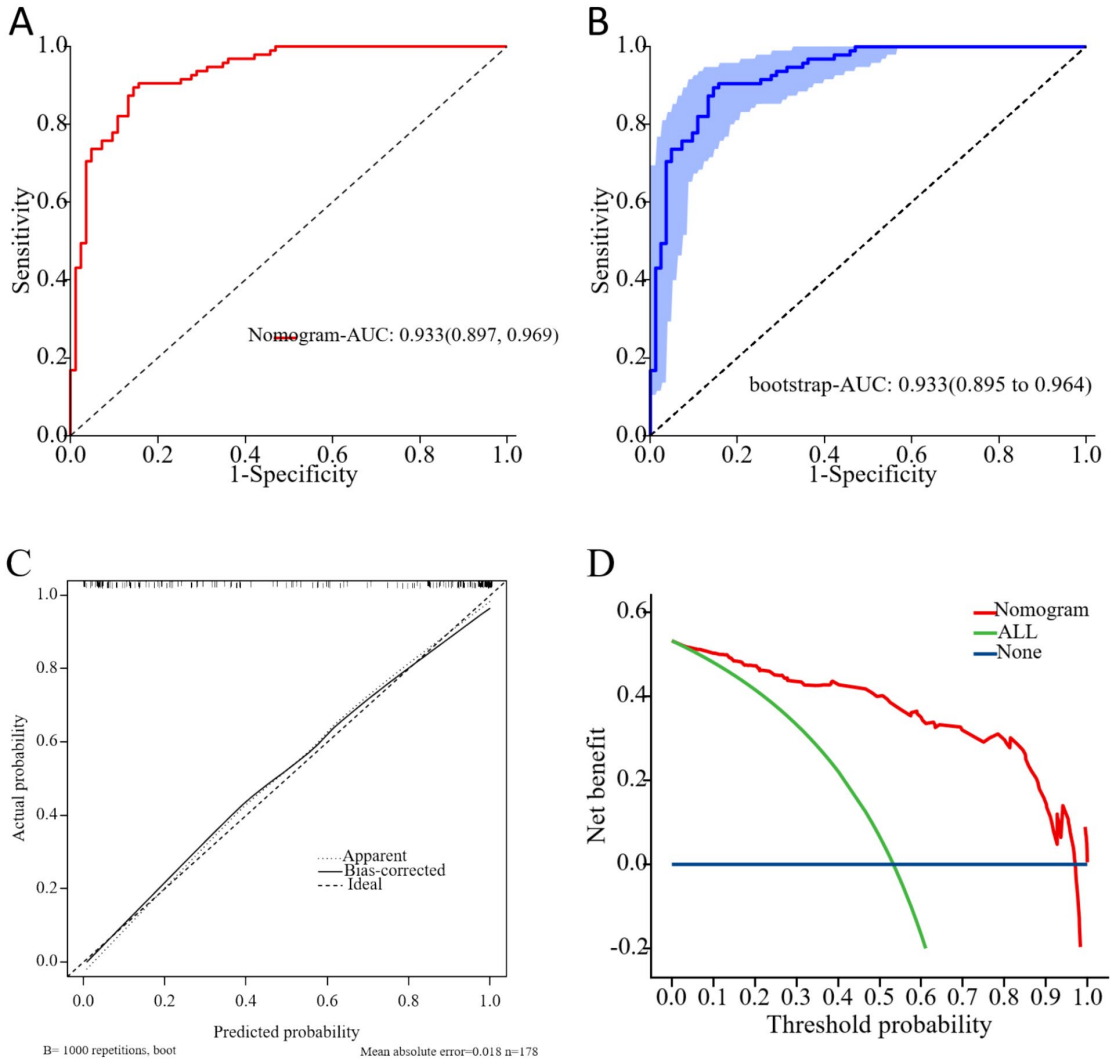
**(A)** Receiver operating characteristic (ROC) curve of nomogram model in predicting the efficacy of 3-month unfavorable outcome in patients with Elderly NVAF - AIS. **(B)** ROC curve of nomogram model in predicting the efficacy of 3-month unfavorable outcome in patients with Elderly NVAF-AIS. (validation sample with 1,000 bootstrap resamples). **(C)** Calibration curve of validation sample for nomogram models. **(D)** Clinical decision curve analysis (DCA) of validation sample for nomogram models.

To prevent overfitting, bootstrap resampling with 1,000 iterations was performed for internal validation. The results of the ROC curve analysis indicate that the AUC values, both prior to and following internal validation of the nomogram, were 0.933 (95% CI: 0.897–0.969) (Figure 4A) and 0.933 (95% CI: 0.895–0.964) (Figure 4B), respectively. Meanwhile, the sensitivity values before and after internal validation were 0.895 and 0.905, specificity values were 0.855 and 0.831, positive predictive values were 0.876 and 0.860, negative predictive values were 0.877 and 0.885, and accuracy rates were 0.876 and 0.871. These results indicated good discriminative performance of the nomogram. The deviation between observed and predicted outcomes was not statistically significant, as confirmed by the Hosmer-Lemeshow test (χ^2^ = 3.616, *p* = 0.306). Similarly, the average absolute error for the model calibration curve was 0.018, and the predicted probabilities closely matched the actual probabilities (Figure 4C). These results suggest high calibration and accuracy for the nomogram.

DCA was plotted with threshold probability on the x-axis and net clinical benefit on the y-axis (Figure 4D). The blue horizontal line represents “None,” where no intervention is applied to any patient, resulting in a net benefit of 0. The green diagonal line represents “All,” where all patients are treated, and the net benefit curve has a negative slope. The red curve represents the DCA curve of the predictive model. As shown in the figure, the DCA curve lies above the “None” and “All” ineffective lines within the x-axis range of 0.05–0.97. This indicates that, within this range, the model demonstrates a favorable performance, confirming its high clinical utility in predicting outcomes for NVAF-AIS patients.

## Discussion

AF is a common arrhythmia and a major risk factor for IS, especially in the elderly. The risk of stroke in patients with NVAF has been reported to increase by 4–5 times (4, 5). Cardiogenic brain infarctions are generally more severe than those caused by other factors, and they are associated with worse prognosis, higher recurrence rates, and increased mortality (6). Since prognosis related to NVAF-AIS is inextricably linked with the disease severity, identification of predictors for outcome and establishment of a clinical prediction model form one major cornerstone for making informed decisions of therapies in elderly patients with NVAF-AIS. Thus, the LASSO regression model was used in the present study to incorporate all the data from demographic, clinical, laboratory, and imaging features without overfitting and skewing its distribution. In the multivariate logistic regression analysis, independent predictors were determined and a predictive nomogram was generated. A predictive nomogram incorporating stroke history, NIHSS score, HbA1c, D-dimer, NLR, and LAD showed good performance regarding discrimination, calibration, and clinical applicability.

The NIHSS score has been generally recognized as an independent predictor of short- and long-term adverse outcomes following stroke and atrial fibrillation (16, 17). Generally, the higher the NIHSS score, representing larger infarct volumes and significant brain edema, poorer the patient outcomes it usually foretells (18, 19). This scoring system is integral to assessing the severity of ischemic stroke and forecasting patient prognoses. In this study, NIHSS was confirmed as an independent risk factor for poor outcomes in NVAF-AIS patients, which is in agreement with previous studies (OR = 1.271, 95% CI: 1.109–1.500). The baseline NIHSS scores in the nomogram remarkably increased its predictive precision. Moreover, a history of stroke was also confirmed as an independent risk factor for acute ischemic stroke in NVAF patients (20, 21). This may be due to more serious intracranial stenosis and lower compensatory capacity of collateral circulation in this population, which is closely related to the recurrence, prognosis, and mortality of AIS. Many literature reports have supported this result (22, 23). In this study, stroke history was also identified as an independent risk factor for poor outcomes in NVAF-AIS patients (OR = 7.441, 95% CI: 2.567–23.860). It is hypothesized that with each additional stroke episode, patients may experience worse outcomes. However, further validation in future studies is necessary.

HbA1c has been shown to correlate with poor functional outcomes after stroke onset in patients with chronic hyperglycemia (24, 25). Elevated levels of reactive oxygen species (ROS) in this population, often due to factors like intracellular acidosis, a procoagulant state, endothelial dysfunction, or hyperglycemia (26, 27), are known to exacerbate brain and reperfusion injuries, thus contributing to worse outcomes. High HbA1c levels, often indicative of an unhealthy lifestyle, limited awareness of associated vascular risk factors, or poor medication adherence, were also observed. HbA1c reflects the average blood glucose level over the 2–3 months prior to and following the onset of AIS. It is less influenced by single glucose fluctuations and therefore has higher predictive value than a single glucose measurement. Research has shown that elevated HbA1c is an independent risk factor for hemorrhagic transformation of AIS induced by NVAF (28). Consistent with these findings, this study found that high HbA1c levels were closely correlated with poor outcomes at 3 months in elderly patients with NVAF-AIS, independent of transient glucose increases upon admission. For patients with cardiogenic stroke, tight glucose control is crucial in reducing the risk of poor outcomes after stroke onset.

In this study, D-dimer levels were notably higher in the poor outcome group, underscoring its role as an independent predictor of adverse outcomes. Literature, including findings by Wang et al. (29), supports the association of elevated serum D-dimer levels with severe neurological impairment, early neurological deterioration, and overall poorer outcomes in patients with acute cerebral infarction. Multiple studies have also shown that admission plasma D-dimer levels significantly correlate with infarct volume and functional outcomes after cardiogenic embolic stroke in NVAF patients (30, 31). D-dimer, a fibrin degradation product, is significantly associated with thrombosis in patients with acute cardiogenic brain embolism. Elevated serum D-dimer indicates thrombus formation in the atria and possibly in cerebral vessels (32). Furthermore, D-dimer elevation can trigger the secretion of pro-inflammatory cytokines from monocytes, leading to an inflammatory response. Activated coagulation and inflammation exert synergistic effects, ultimately contributing to stroke development (33, 34). Therefore, D-dimer levels have important implications for the diagnosis, efficacy assessment, and prognosis of thrombotic diseases. Additionally, this study validated that elevated NLR significantly distinguished the poor outcome group from their counterparts. Logistic regression verified NLR as an independent risk factor for adverse outcomes in elderly NVAF-AIS patients.

Logistic regression analysis indicated that NLR was an independent risk factor for bad outcomes in older patients with NVAF-AIS, and the study found a statistically significant increase in NLR in the poor outcome group compared to the good outcome group. NLR is a simple clinical biomarker that is easily accessible and can reliably predict 3-month functional outcomes in stroke patients (35, 36). An observational study revealed a close relationship between NLR and hospital mortality as well as NIHSS score in patients with NVAF-AIS (37). Currently, NLR is used as a biomarker for the prognosis of various inflammatory diseases, including cerebrovascular and coronary artery diseases (38). Multiple mechanisms underlie these associations. Neutrophils secrete large amounts of inflammatory mediators, leading to severe endothelial cell dysfunction, axonal injury, and increased blood–brain barrier permeability. These changes exacerbate neural injury. On the other hand, regulatory T and B cells, lymphocytes with neuroprotective properties, exert regulatory functions in AIS, reducing ischemic brain tissue damage and improving neurological deficits. Neutrophils and lymphocytes play important but distinct roles. Neither neutrophils nor lymphocytes alone have strong clinical utility, as their associations may be overlooked. Incorporating NLR, a potential biomarker for cerebrovascular events in AF patients, into our model is essential.

Increased LAD was also identified as an independent risk factor for poor outcomes in patients with NVAF-AIS in this study. Left atrial abnormality is a well-known marker of structural heart disease, left ventricular pressure, and volume overload (39). It is also independently associated with AF, cardiogenic embolic stroke, and all-cause mortality (40–43). Xue et al. found that left atrial enlargement correlated with more severe neurological deficits in patients with cardiogenic embolism (44). As left atrial volume increases, the blood flow speed within the left atrial appendage (which contains about 90% of thrombi in the left atrium) decreases, increasing the risk of blood stasis and thrombus formation. Moreover, the study suggested that the severity of ischemic stroke could correlate with the size of the left atrial appendage. Enlargement of the left atrium is a well-established risk factor for atrial fibrillation, often leading to large vessel occlusion in stroke. Research indicates that acute stroke patients with large vessel occlusion and left atrial enlargement face a doubled risk of poor collateral circulation compared to those with normal atrial dimensions (45). This finding suggests that left atrial abnormality may increase stroke risk and lead to poor outcomes. Endothelial dysfunction is known to be associated with both left atrial enlargement and poor collateral circulation, but further research is needed.

This study has several advantages. First, participants were confined to elderly patients diagnosed with NVAF-AIS, and the model could be tailor-made for this group. Second, the nomogram combined many clinically important risk factors; thus, it could enable precise calculations of the probability of every clinical event. Third, further addition of some very valuable predictors, like serum inflammatory markers, improves the reliability and robustness of the model. Fourth, all the variables included in the nomogram were easily accessible and important to understand when it came to poor outcome prediction among this population.

However, there are several limitations in this study. First, the sample size was small; more samples should be added in further studies to raise the accuracy. Second, as a single-center retrospective study, some bias may exist. Besides, external validation in other stroke centers is needed because of the difference in races, economic conditions, hospitals, and treatment protocols. Third, there is a lack of the inclusion of magnetic resonance imaging and computed tomography angiography data in this study, which could weaken the scientific rigor in this model. Despite such limitations, the model established in this study might be of clinical interest.

In conclusion, a nomogram consisting of stroke history, NIHSS score, HbA1c, D-dimer, NLR, and LAD was developed to predict 3-month outcomes in elderly patients with NVAF-AIS. However, further external validation is essential to assess the broader applicability of this model. Additionally, incorporating machine learning techniques could enhance the accuracy of future prognostic predictions, while dynamic monitoring of laboratory indicators may further refine the precision of long-term prognosis assessments for elderly patients with acute cardioembolic stroke. Such advancements would be beneficial for improving long-term rehabilitation outcomes for these patients.

## Data Availability

The original contributions presented in the study are included in the article/supplementary material, further inquiries can be directed to the corresponding author.
